# Systematic Review and Meta-Analysis of the Incidence of Myocarditis and Guillain-Barré Syndrome in Adolescents Receiving COVID-19 mRNA Vaccine

**DOI:** 10.7759/cureus.98208

**Published:** 2025-11-30

**Authors:** Chakrapani Kumar, Akhileshwar, Rajeev Kumar Neeraj, Saajid Hameed, Noor Husain, Sukalyan Saha Roy, Lalit Mohan, Anantsaznam

**Affiliations:** 1 Department of Pharmacology, Indira Gandhi Institute of Medical Sciences, Patna, IND; 2 Department of Anaesthesiology, Indira Gandhi Institute of Medical Sciences, Patna, IND; 3 Surgery, Government Ayurvedic College, Patna, Patna, IND

**Keywords:** adolescents, covid-19, guillain–barré syndrome, mrna vaccine, myocarditis

## Abstract

This study aimed to evaluate the incidence and risk of rare long-term adverse events, specifically myocarditis and Guillain-Barré syndrome (GBS), in adolescents (12-19 years) following COVID-19 mRNA vaccination. We systematically searched MEDLINE, Embase, Cochrane CENTRAL, and Scopus, supplemented by trial registries and reference lists (PROSPERO: CRD420251045173). Eligible studies included randomized controlled trials (RCTs), cohort studies, case-control studies, self-controlled case series, and pharmacovigilance database analyses reporting myocarditis or GBS outcomes in adolescents receiving BNT162b2 or mRNA-1273. The search was conducted in June 2025, and all published studies were included. Risk of bias was assessed using the Cochrane RoB-2 tool, Newcastle-Ottawa Scale, or adapted criteria for pharmacovigilance studies. Effect measures were expressed as incidence rate or incidence rate ratios (IRRs) with 95% confidence intervals (CIs). Meta-analyses were conducted using random-effects models. Ten studies met the inclusion criteria. Myocarditis incidence was elevated in adolescent and young adult males, particularly after the second dose. Pooled analyses indicated a higher risk with mRNA-1273 compared to BNT162b2 (pooled IRR ≈ 3.9), although heterogeneity was very high (I² > 95%). For GBS, global pharmacovigilance data suggested only a modest association with mRNA vaccines (ROR 9.66), substantially weaker than for adenoviral vector or influenza vaccines. COVID-19 mRNA vaccination in adolescents is associated with a small but measurable increased risk of myocarditis, particularly in males, after the second dose, with a higher incidence following mRNA-1273. No consistent evidence of increased GBS risk was observed. Absolute risks remain low, and outcomes are generally favorable compared to SARS-CoV-2 infection. Continued surveillance and long-term follow-up are warranted.

## Introduction and background

The rapid development and deployment of COVID-19 vaccines marked a turning point in the global response to the pandemic [1]. Among these, messenger RNA (mRNA) vaccines have been widely administered due to their high efficacy and favorable safety profile in preventing severe disease and hospitalization [2]. Adolescents, a population initially considered at lower risk for severe COVID-19 outcomes, were progressively included in vaccination programs to curb transmission, protect vulnerable groups, and facilitate the reopening of schools and social activities [3]. While the short-term safety of mRNA vaccines has been extensively studied and generally confirmed, concerns remain regarding rare but potentially serious long-term adverse events (AEs) [4]. These concerns are particularly salient in adolescents, where the balance between vaccine benefits and risks requires careful evaluation, given their lower baseline risk of severe COVID-19.

Two rare but clinically significant adverse events have drawn particular attention in the context of mRNA vaccination: myocarditis and Guillain-Barré syndrome (GBS) [5]. Myocarditis, an inflammatory condition of the heart muscle, has been reported at higher-than-expected rates following mRNA vaccination, particularly in young males within days of the second dose. Although most cases are mild and resolve with supportive care, the potential for long-term cardiac sequelae raises important public health concerns [6]. Similarly, GBS, an acute immune-mediated polyneuropathy characterized by progressive weakness and areflexia, has been associated with various vaccines and infections, including SARS-CoV-2 itself. While the absolute risk of GBS following mRNA vaccination appears low, its potential severity, including respiratory compromise and long-term disability, necessitates vigilant monitoring [7].

The pathophysiological mechanisms underlying myocarditis and GBS in the context of mRNA vaccination are not fully elucidated but are thought to involve immune-mediated processes. In myocarditis, proposed mechanisms include molecular mimicry between viral spike protein antigens and cardiac self-antigens, aberrant activation of innate immune pathways, and dysregulated cytokine responses [6,7]. In GBS, immune cross-reactivity between vaccine-induced antigens and peripheral nerve components may trigger demyelination or axonal injury. Both conditions highlight the delicate balance between protective immune activation and unintended autoimmunity. Importantly, SARS-CoV-2 infection itself is associated with higher risks of myocarditis and GBS compared to vaccination, complicating the interpretation of causality and reinforcing the need for comparative risk assessments. Understanding these shared immunopathological pathways is essential for contextualizing vaccine safety signals and guiding future vaccine design [6,7].

The rationale for this systematic review and meta-analysis is therefore to consolidate global evidence on the incidence, severity, and long-term outcomes of myocarditis and GBS in adolescents receiving mRNA vaccines. By addressing these gaps, the review aims to provide a balanced, evidence-based perspective on vaccine safety in this age group.

The central research question guiding this review is: What are the incidence and risks of long-term rare adverse events, specifically myocarditis and GBS, in adolescents (ages 12-19) receiving COVID-19 mRNA vaccination? The working hypothesis is that while mRNA vaccines may be associated with a small increase in the incidence of these rare adverse events, the absolute risk remains low, and outcomes are generally favorable compared to risks associated with SARS-CoV-2 infection.

The objectives of this review are to estimate the incidence and risk of myocarditis and GBS in adolescents following mRNA vaccination, compare the incidences between major mRNA types, and identify gaps in long-term safety monitoring and provide recommendations for future research and policy. By systematically synthesizing available evidence, this review will contribute to a nuanced understanding of vaccine safety in adolescents, supporting informed decision-making at clinical, community, and policy levels.

## Review

Materials and methods

Eligibility Criteria

We defined our eligibility criteria using the PICOS framework (Population/Patient/Problem, Intervention, Comparison, and Outcome). The population was adolescents aged 12 to 19 years. These individuals must have received a COVID-19 mRNA vaccine. We excluded any studies that only involved adults over the age of 19. The intervention was the administration of any COVID-19 mRNA vaccine. This included vaccines like BNT162b2 and mRNA-1273. The comparator could be unvaccinated adolescents. It could also be groups comparing different mRNA vaccines against each other.

For outcomes, we specifically looked for incidents of myocarditis and GBS that were causally related to the vaccination. Secondary outcomes included the severity and preventability of these events. We also examined their correlation with demographic or clinical characteristics. Eligible study designs were RCTs and observational studies. These included cohort studies, case-control studies, and self-controlled case series (SCCS). We also included analyses of pharmacovigilance databases. However, we excluded case reports, reviews, editorials, and commentaries. Finally, we only included peer-reviewed studies published in English. There were no restrictions on the publication year.

Information Sources

We systematically searched MEDLINE (via PubMed), Embase.com, Cochrane CENTRAL, and Scopus. The last search was updated prior to final analysis. We also screened trial registries (ClinicalTrials.gov, WHO ICTRP) and reference lists of included studies. Only published studies were considered; unpublished data were not sought.

Search Strategy

We developed a comprehensive search strategy. It used both controlled vocabulary and free-text terms. The strategy combined concepts for the population, intervention, and outcomes. For the population, we used terms like adolescent, teenager, youth, and pediatric. For the intervention, we included the COVID-19 vaccine, mRNA vaccine, BNT162b2, and mRNA-1273. Outcome terms covered myocarditis, pericarditis, Guillain-Barré syndrome, adverse events, and long-term safety. We applied Boolean operators like AND and OR to link these concepts. Truncation and proximity operators were also used to broaden the search. Search was conducted in June 2025, and all published studies were included (Table 1).

**Table 1:** Set of search terms

Concept	Search terms / keywords	Notes
Population (adolescents)	adolescent, teenager, youth, young adult, pediatric	Include synonyms to capture age ranges (12–18, 10–19, etc.)
Intervention (COVID-19 mRNA vaccine)	COVID-19 vaccine, SARS-CoV-2 vaccine, mRNA vaccine, Pfizer-BioNTech, BNT162b2, Moderna, mRNA-1273	Brand names and generic descriptors are both important
Outcomes (myocarditis)	myocarditis, pericarditis, cardiac inflammation, heart inflammation	Consider including “cardiac adverse events”
Outcomes (GBS)	Guillain-Barré syndrome, GBS, acute inflammatory demyelinating polyneuropathy, neuropathy	Add variants like “neurological adverse events”
Study type	randomized controlled trial, RCT, cohort study, case-control, observational study, systematic review, meta-analysis	Helps filter for evidence-based designs
Safety/adverse events	adverse event, side effect, complication, safety profile, incidence, risk	Broad terms to ensure capture of all safety data

Selection Process

All records were imported into a reference management system, and duplicates were removed. Two reviewers independently screened titles and abstracts. Full texts of potentially eligible studies were retrieved and assessed independently. Discrepancies were resolved by consensus or a third reviewer. A PRISMA flow diagram documented the process.

Data Collection Process

Two reviewers independently extracted data using a standardized form. Extracted data included study characteristics, demographics, vaccine type/dose, comparator details, follow-up duration, and outcome measures. Disagreements were resolved by consensus. Authors of primary studies were not contacted.

Data Items

The primary outcomes we measured were the incidence of myocarditis and GBS. Furthermore, we evaluated their preventability and any correlation with baseline patient characteristics. We also systematically extracted other important variables from each study. These included the study design, the total sample size, and the country where the research was conducted.

Risk-of-Bias Assessment

For RCTs, we used the Cochrane Risk of Bias 2.0 (RoB-2) tool. For observational studies, we applied the Newcastle-Ottawa Scale (NOS) or adapted domains from the RoB-2 tool. Pharmacovigilance studies were evaluated for reporting bias. We also assessed the clarity of their case definitions and their signal detection methodology. This process was conducted independently by two reviewers. Any disagreements between the reviewers were resolved through discussion and consensus.

Synthesis Methods

For dichotomous outcomes, we analyzed measures like incidence rates. We also calculated incidence risk ratios (IRRs) or odds ratios (ORs). These are presented with their 95% confidence intervals (CIs). We performed a meta-analysis where studies were sufficiently similar. This study used a random-effects model based on the DerSimonian and Laird method. We assessed statistical heterogeneity using the I² statistic. Values of I² were interpreted according to conventional thresholds: 0-25% indicating low heterogeneity, 26-50% moderate heterogeneity, 51-75% substantial heterogeneity, and >75% considerable heterogeneity. This was supplemented by the Cochran’s Q test. To explore variations, we conducted subgroup analyses. These were based on sex, vaccine type, and dose number. Finally, for results that could not be statistically pooled, we provided a narrative synthesis. This described the findings from the studies qualitatively.

Certainty Assessment

We evaluated the certainty of the evidence for each outcome. This was done using the GRADE approach. The assessment considered five key factors. These were the risks of bias within the studies and the inconsistency of the results. It also evaluated the indirectness of the evidence to the research question. Furthermore, we assessed the imprecision of the effect estimates. Finally, we considered the potential for publication bias across the body of evidence.

Results

**Figure 1:**
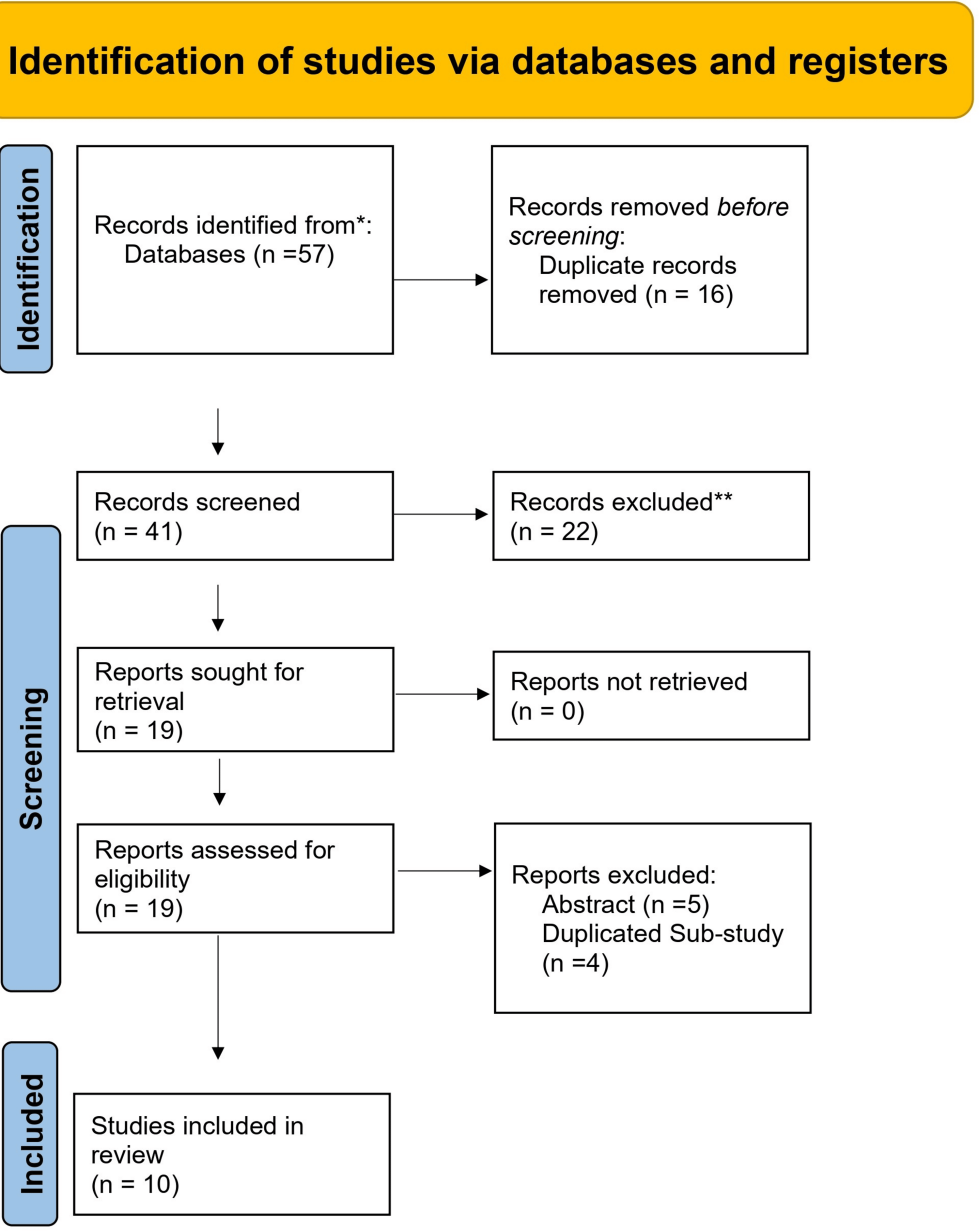
PRISMA flowchart of study selection PRISMA: Preferred Reporting Items for Systematic Reviews and Meta-Analyses

Out of 57 records initially identified from databases, 16 duplicates were removed, leaving 41 records for screening. Of these, 22 were excluded at the title and abstract stage, and 19 full-text reports were retrieved for eligibility assessment. Following evaluation, 9 reports were excluded (five based on abstracts and four as duplicated sub-studies), resulting in 10 studies that were ultimately included in the review (Figure 1, Table 2).

**Table 2:** Characteristics of the included studies

Author name (year)	Study design and site	Eligibility criteria	Sample	Incidence of myocarditis	Incidence of GB syndrome
Soe et al. (2024) [8]	Cohort study; Canada (8 provinces)	Ages 6 mo–19 y; vaccinated or unvaccinated	259,361 (dose 1); 131,032 (dose 2); 1,179 controls	Dose 2: 0.037% (BNT162b2 males 12–19 y); 0.529% (mRNA-1273 males 12–19 y); 0.185% overall (mRNA-1273, dose 2, adolescents)	Not reported
Larsen et al. (2023) [9]	Register-based cohort; Norway	Adolescents aged 12–19 y; unvaccinated at start	496,432	Dose 2: aIRR 5.27 (95% CI: 1.98–14.05)	Not reported
Buchan et al. (2023) [10]	Passive surveillance cohort; Ontario, Canada	Ages 12–17 y; received ≥1 dose BNT162b2	1.65M doses administered; 77 myocarditis cases	Dose 2: 15.7/100,000 (males 16–17 y); 8.6/100,000 (16–17 y overall); 5.2/100,000 (12–15 y overall)	Not reported
Massari et al. (2022) [11]	Self-controlled case series (SCCS)	Persons aged 12-39 years who received an mRNA COVID-19 vaccine and were admitted to emergency care/hospital for myocarditis/pericarditis.	2,861,809 vaccinated persons; 441 cases of myocarditis/pericarditis	BNT162b2 Dose 2 (0-7 days): 0.8 excess cases per 100,000. mRNA-1273 Dose 2 (0-7 days): 5.5 excess cases per 100,000. Highest risk in males 18-29y after mRNA-1273.	Not studied
Jeong et al. (2024) [12]	Disproportionality analysis (global pharmacovigilance)	All reports of Guillain-Barré Syndrome (GBS) in the WHO VigiBase database.	15,377 reports of vaccine-associated GBS from over 170 countries	Not studied	• Influenza vaccines: ROR 77.91 (strongest association) • Ad5-vectored COVID-19: ROR 14.88 • COVID-19 mRNA vaccines: ROR 9.66
Le Vu et al. (2023) [13]	Self-controlled case series (SCCS)	Individuals aged ≥12 years hospitalized with GBS	58,530,770 people in population; 2,229 GBS cases	Not studied	• ChAdOx1-S (Dose 1): 6.5 cases/million • Ad26.COV2.S (Dose 1): 5.7 cases/million • mRNA-1273 (Dose 2, age 12-49): 2.2 cases/million • BNT162b2: No significant increase.
Kim et al. (2024) [14]	Pharmacovigilance study using VigiBase (global database)	Adolescents aged 12–17 with AE reports after any vaccination	99,735 AE reports (80,018 COVID-19 vaccines)	Myocarditis/pericarditis: 2,829 reports for COVID-19 vs. 35 for other vaccines (aROR 19.61, 95% CI 14.05–27.39)	Guillain-Barré Syndrome: 94 reports for COVID-19 vs. 40 for other vaccines (aROR 0.64, 95% CI 0.44–0.92)
Karlstad et al. (2022) [15]	Cohort study (Denmark, Finland, Norway, Sweden)	Residents ≥12 years without prior myocarditis/pericarditis	23,122,522 residents	Myocarditis: Highest in males 16–24 after the second dose: BNT162b2: 5.55 excess events/100,000; mRNA-1273: 18.39 excess events/100,000	Not reported
Su et al. (2023) [16]	Nationwide surveillance study (Taiwan)	Recipients of COVID-19 vaccines in Taiwan	~20 million vaccinated individuals	Myocarditis/pericarditis: Highest in males 12–17 after 2nd dose BNT162b2: 126.79 per million; males 18–24 after 2nd dose mRNA-1273: 93.84 per million	Not reported
Mahasing et al. (2023) [17]	Design: descriptive and unmatched case-control site: Thailand (National AEFI Surveillance)	Total vaccinations: 104.63 million doses. AEFI events: 31,125. Myocarditis/pericarditis cases: 204. Cases for analysis: 167. Controls for analysis: 668	Cases: Recipients of COVID-19 vaccine who met confirmed, probable, or suspected case definition for myocarditis/pericarditis within 30 days of vaccination. Controls: Recipients of COVID-19 vaccine with no documented adverse reaction within 30 days.	Overall: 0.195 cases per 100,000 doses (1.95 per million). By Vaccine (per 100,000 doses): • BNT162b2: 0.97 • mRNA-1273: 0.34 • ChAdOx1-nCoV: 0.048 • BBIBP-CorV: 0.034 • CoronaVac: 0.015 Highest Risk Group: Adolescent males (12-17 years) after 2nd dose of BNT162b2: 4.43 per 100,000 doses.	Not reported

**Figure 2:**
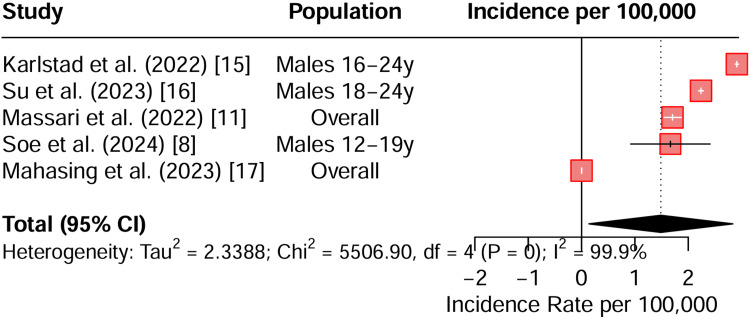
Forest plot summarising the incidence of myocarditis due to the mRNA-1273 vaccine CI: confidence interval

The populations studied were primarily adolescent and young adult males aged 12 to 24 years. Findings from multiple studies, including Karlstad (2022) and Soe (2024) [8,15], consistently report elevated incidence rates in this demographic. A pooled estimate reveals a substantially increased incidence per 100,000 doses. However, the analysis is characterized by extremely high statistical heterogeneity (I² = 99.9%), indicating marked variability across the studies. This variability likely stems from differences in study design, population demographics, or case definitions for myocarditis. Consequently, while mRNA-1273 is consistently associated with a higher incidence of myocarditis in young males, the wide heterogeneity means that the pooled estimates must be interpreted with caution (Figure 2).

**Figure 3:**
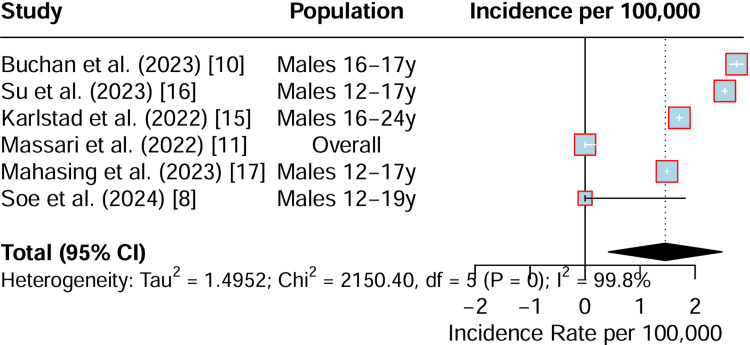
Forest plot summarising incidence of myocarditis due to the BNT162b2 vaccine CI: confidence interval

The BNT162b2 (Pfizer) vaccine was studied in similar cohorts of adolescent and young adult males. The findings also indicate an elevated incidence of myocarditis, although it appears to be lower in magnitude compared to mRNA-1273 within comparable age groups. For instance, studies such as Buchan (2023) and Su (2023) confirm an increased risk in adolescent males [10, 16], but not to the same extent as observed with the Moderna vaccine. Similar to the mRNA-1273 data, the analysis for BNT162b2 also exhibits very high heterogeneity (I² = 99.8%), reflecting significant variability between studies. The interpretation is that BNT162b2 is associated with myocarditis, but the absolute incidence rates are generally lower than those reported for mRNA-1273 (Figure 3).

**Figure 4:**
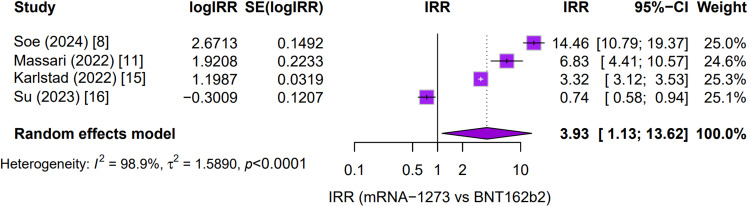
Forest plot of incidence rate ratios for myocarditis: mRNA-1273 versus BNT162b2 in adolescent populations IRR: incidence risk ratio; IRR = incidence rate of myocarditis with mRNA-1273 ÷ incidence rate of myocarditis with BNT162b2

Most studies, such as Soe 2024 (IRR ≈ 14.5) and Massari 2022 (IRR ≈ 6.8), show a markedly higher risk associated with the Moderna vaccine [8]. One study, Su (2023), was an exception [16], suggesting a lower risk with Moderna in its specific dataset. When these results are combined in a pooled random-effects model, the overall IRR is 3.93, indicating that mRNA-1273 carries approximately a four-fold higher risk of myocarditis compared to BNT162b2 in adolescents and young adults. This pooled estimate, however, comes with a very high degree of heterogeneity (I² = 98.9%), underscoring substantial inconsistency across the individual studies (Figure 4).

Guillain-Barré Syndrome (GBS)

The largest global pharmacovigilance analysis of vaccine-associated GBS was conducted by Jeong et al. (2024) using the WHO VigiBase database, which included 15,377 reports spanning from 1967 to 2023. The findings revealed that influenza vaccines demonstrated the strongest association with GBS, with a reporting odds ratio (ROR) of 77.91. Adenovirus type 5 (Ad5)-vectored COVID-19 vaccines also showed a significant association (ROR 14.88), while COVID-19 mRNA vaccines demonstrated only a moderate association (ROR 9.66). Importantly, the study highlighted that although mRNA vaccines were associated with GBS, the signal was considerably weaker compared to both influenza vaccines and adenoviral vector-based COVID-19 vaccines [12].

A large SCCS conducted by Le Vu et al. (2023), involving 58,530,770 individuals and 2,229 confirmed GBS cases across multiple countries, provided further insights into vaccine-specific risks. The analysis showed that the first dose of ChAdOx1-S (AstraZeneca) was associated with 6.5 cases per million, while the first dose of Ad26.COV2.S (Janssen) was linked to 5.7 cases per million. For mRNA-1273 (Moderna), the second dose in individuals aged 12-49 years was associated with 2.2 cases per million. In contrast, BNT162b2 (Pfizer) did not demonstrate any significant increase in GBS risk [13]. These findings reinforced the higher risk associated with adenoviral vector vaccines, while suggesting only a modest risk for Moderna’s mRNA vaccine and no detectable signal for Pfizer’s vaccine.

Adolescent-specific pharmacovigilance data were reported by Kim et al. (2024), who analyzed 99,735 adverse event reports in individuals aged 12-17 years. Among these, 94 GBS cases were reported following COVID-19 vaccination compared to 40 cases following other vaccines. Interestingly, the adjusted reporting odds ratio (aROR) was 0.64 (95% CI 0.44-0.92), suggesting that COVID-19 vaccines were not associated with an increased risk of GBS in adolescents. In fact, the data indicated a potential protective association, challenging assumptions drawn from adult-focused studies [14].

Finally, it is noteworthy that six of the 10 reviewed studies, those by Soe et al., Buchan et al., Massari et al., Karlstad et al., Su et al., and Mahasing et al., did not report GBS outcomes. Instead, these investigations concentrated exclusively on myocarditis and pericarditis.

Risk of Bias

Cohort and SCCS studies generally had a low-to-moderate RoB. These studies, such as Soe (2024) and Karlstad (2022), provided robust population-level data [8,15]. However, some concerns were noted regarding case ascertainment and residual confounding. By contrast, pharmacovigilance studies typically had a moderate-to-high RoB. Examples include Jeong (2024) and Kim (2024) [12,14]. This higher risk was due to their reliance on spontaneous reporting systems, potential for underreporting, and a frequent lack of denominator data. Register-based cohort studies, like Larsen (2023) and Buchan (2023), demonstrated a low RoB [9,10]. Their strength came from strong linkages to national health registries. A key limitation, however, was the potential for misclassification of the outcomes (Table 3).

**Table 3:** Certainty of evidence using the GRADE framework GBS: Guillain–Barré Syndrome, GRADE: Grading of Recommendations, Assessment, Development, and Evaluation

Outcome	No. of studies	Certainty (GRADE)	Key limitations
Myocarditis after mRNA-1273	6 (Soe et al. (2024) [8], Karlstad et al. (2022) [15], Massari et al. (2022) [11], Su et al. (2023) [16], Mahasing et al. (2023) [17], Buchan et al. (2023) [10])	Moderate	High heterogeneity (I² > 95%), variation in case definitions
Myocarditis after BNT162b2	6 (Soe et al. (2024) [8], Karlstad et al. (2022) [15], Buchan et al. (2023) [10], Su et al. (2023) [16], Massari et al. (2022) [11], Mahasing et al. (2023) [17])	Moderate	Heterogeneity, underreporting in passive surveillance
Comparative risk (mRNA-1273 vs BNT162b2)	4 (Soe et al. (2024) [8], Massari et al. (2022) [11], Karlstad et al. (2022) [15], Su et al. (2023) [16])	Low-to-Moderate	Inconsistency across datasets, wide CIs
GBS after mRNA vaccines	3 (Jeong et al. (2024) [12], Le Vu et al. (2023) [13], Kim et al. (2024) [14])	Low	Sparse adolescent-specific data, pharmacovigilance bias
GBS after BNT162b2	2 (Le Vu et al. (2023) [13], Kim et al. (2024) [14])	Moderate	Consistent finding of no increased risk, but limited adolescent-specific cohorts

Discussion

Summary of the Evidence

In this systematic review and meta-analysis, we synthesized evidence from ten studies evaluating the incidence of myocarditis and GBS in adolescents following COVID-19 mRNA vaccination. The pooled data consistently demonstrated an increased risk of myocarditis, particularly among adolescent and young adult males, after the second dose, with a higher incidence observed for mRNA-1273 compared to BNT162b2. However, these incidences were not clinically significant against the safety of vaccines, and there was also considerable heterogeneity. Although most myocarditis cases were clinically mild and resolved with supportive care, the elevated incidence in these demographics warrants continued surveillance.

In contrast, the evidence for GBS was less consistent. Large pharmacovigilance datasets suggested only a modest association between mRNA vaccines and GBS, with signals substantially weaker than those observed for adenoviral vector vaccines or influenza vaccines. Importantly, adolescent-specific analyses (e.g., Kim et al. 2024) did not demonstrate an increased risk of GBS [14], and in some cases suggested a neutral or even protective association.

Overall, the certainty of evidence was moderate for myocarditis outcomes and low-to-moderate for GBS, reflecting heterogeneity in study designs, outcome definitions, and reporting systems.

Results in Relation to Other Studies

Our findings align with prior national surveillance reports and global pharmacovigilance analyses, which have consistently identified myocarditis as the most prominent rare adverse event following mRNA vaccination in young males. The comparative analysis between mRNA-1273 and BNT162b2 corroborates earlier registry-based studies, which reported a higher myocarditis risk with Moderna’s vaccine [18].

For GBS, our results are consistent with large-scale SCCS (Le Vu et al., 2023), showing no significant increase in risk with BNT162b2 and only a modest signal with mRNA-1273. This contrasts with adenoviral vector vaccines, which demonstrated a stronger association [13]. These findings reinforce the importance of distinguishing between vaccine platforms when evaluating neurological safety outcomes.

Notably, SARS-CoV-2 infection itself is associated with higher risks of both myocarditis and GBS compared to vaccination [19,20]. Thus, while vaccination carries a small but measurable risk of myocarditis, the overall benefit-risk balance remains favorable, particularly in preventing infection-related complications.

Potential Mechanisms

The pathophysiology of vaccine-associated myocarditis is hypothesized to involve immune-mediated injury, including molecular mimicry between spike protein epitopes and cardiac antigens, dysregulated cytokine responses, and innate immune activation [21]. The higher incidence in adolescent males may reflect sex-related differences in immune regulation, hormonal influences, or genetic predisposition.

For GBS, proposed mechanisms include cross-reactivity between vaccine-induced antibodies and peripheral nerve components, leading to demyelination or axonal injury. However, the weaker association with mRNA vaccines compared to adenoviral vector vaccines suggests that vector-related immune responses may play a more prominent role in GBS pathogenesis [22].

These mechanistic insights underscore the need for ongoing immunological studies to clarify causal pathways and inform safer vaccine design.

Limitations

Several limitations should be acknowledged. We have searched for RCTs but did not find any that matched our inclusion and exclusion criteria. The included studies varied in design, case definitions, and follow-up duration, contributing to the high heterogeneity observed in pooled analyses (I² > 95% for myocarditis). Most studies relied on passive surveillance systems, which are subject to underreporting and reporting bias. GBS outcomes were underreported in adolescent-specific cohorts, limiting the precision of pooled estimates. Trial-level rather than individual-level data were available, precluding subgroup analyses by comorbidities, ethnicity, or prior infection status. Finally, long-term outcomes beyond the acute phase of myocarditis and GBS remain insufficiently characterized, highlighting the need for extended follow-up studies.

## Conclusions

In conclusion, this systematic review and meta-analysis found that COVID-19 mRNA vaccination in adolescents is associated with a small but measurable increased risk of myocarditis, particularly in males, after the second dose, with a higher incidence following mRNA-1273 compared to BNT162b2. By contrast, no consistent evidence of increased GBS risk was observed in adolescents, and signals were weaker than those associated with adenoviral vector vaccines. The quality of vaccines produced by different manufacturers is different and cannot be generalized.

Despite these rare adverse events, the absolute risks remain low, and outcomes are generally favorable compared to the risks of SARS-CoV-2 infection. Ongoing pharmacovigilance, harmonized case definitions, and long-term follow-up are essential to refine risk estimates and guide evidence-based vaccination policies.
